# *Escherichia coli* Mastitis in Dairy Cattle: Etiology, Diagnosis, and Treatment Challenges

**DOI:** 10.3389/fmicb.2022.928346

**Published:** 2022-07-07

**Authors:** Débora Brito Goulart, Melha Mellata

**Affiliations:** ^1^Department of Veterinary Microbiology and Preventive Medicine, Iowa State University, Ames, IA, United States; ^2^Department of Food Science and Human Nutrition, Iowa State University, Ames, IA, United States; ^3^Interdepartmental Microbiology Graduate Program, Iowa State University, Ames, IA, United States

**Keywords:** mammary pathogenic *Escherichia coli*, antimicrobial resistance, virulence, cattle, mastitis pathogenesis, host-pathogen interaction, milk, immune response

## Abstract

Bovine mastitis is an inflammation of the udder tissue parenchyma that causes pathological changes in the glandular tissue and abnormalities in milk leading to significant economic losses to the dairy industry across the world. Mammary pathogenic *Escherichia (E.) coli* (MPEC) is one of the main etiologic agents of acute clinical mastitis in dairy cattle. MPEC strains have virulence attributes to resist the host innate defenses and thrive in the mammary gland environment. The association between specific virulence factors of MPEC with the severity of mastitis in cattle is not fully understood. Furthermore, the indiscriminate use of antibiotics to treat mastitis has resulted in antimicrobial resistance to all major antibiotic classes in MPEC. A thorough understanding of MPEC’s pathogenesis and antimicrobial susceptibility pattern is required to develop better interventions to reduce mastitis incidence and prevalence in cattle and the environment. This review compiles important information on mastitis caused by MPEC (e.g., types of mastitis, host immune response, diagnosis, treatment, and control of the disease) as well as the current knowledge on MPEC virulence factors, antimicrobial resistance, and the dilemma of MPEC as a new pathotype. The information provided in this review is critical to identifying gaps in knowledge that will guide future studies to better design diagnostic, prevent, and develop therapeutic interventions for this significant dairy disease.

## Introduction

Bovine mastitis is an inflammation of the udder tissue parenchyma that may result in physical, chemical, and bacteriological changes in milk, and pathological alterations in the glandular tissue. Although mastitis can be caused by multiple events, e.g., traumatic, physiological, allergic, etc., infectious events are the most prevalent, as more than 140 species of microorganisms are involved in bovine mastitis (Radostitis et al., 2007). The disease results in significant financial losses owing to diagnostic tests, veterinary services, wasted milk and labor, and indirect expenses such as low reproduction rates and premature culling of affected animals (Heikkilä et al., 2018; Romero et al., 2018; Hogeveen et al., 2019). Mastitis-related economic losses in the United States are projected to be USD 2 billion annually (Rollin et al., 2015), with each mastitis case costing producers an average of USD 326 (Liang et al., 2017). In Israel, Holstein cows diagnosed with clinical mastitis caused by *Escherichia coli* had a decrease in daily milk yield of almost 15%, which is considered an average milk loss of about 200 liters per cow during the 305 days of the lactation period (Blum et al., 2014). In persistent infections, the loss of production is significantly higher; it is estimated at 1,500 liters of milk per cow with a decrease in daily production greater than 30% (Blum et al., 2014). In addition to its effects on dairy cattle, mastitis poses a threat to human health due to the risk of transmitting zoonotic pathogens through ingestion of contaminated milk or direct contact with infected cattle (Sugrue et al., 2019; Maity and Ambatipudi, 2021). Importantly, the emergence of antibiotic-resistant bacteria in milk and dairy products due to the indiscriminate use of antibiotics to treat bovine mastitis is a serious concern (Boireau et al., 2018; Singh et al., 2018). Notably, *E. coli* is a leading cause of acute clinical mastitis in dairy cattle worldwide (Shpigel et al., 2008; Blum et al., 2017; Gao et al., 2017).

*Escherichia coli* comprises a highly heterogeneous group of commensal residents of the gut; however, because of the flexibility of its genome (Méric et al., 2016; Blum et al., 2017; Denamur et al., 2021), this organism has evolved into pathogenic strains capable of causing diseases, including bovine mastitis following fecal contamination of the teat skin. Mammary pathogenic *E. coli* (MPEC) has been proposed as a new pathotype responsible for causing mastitis in dairy animals (Shpigel et al., 2008; Richards et al., 2015). The mammary gland is not a natural or primary habitat for *E. coli* because of the milk-associated innate immunity components, such as antimicrobial peptides, lysozyme, lactoferrin, and complement (Alamdari and Ehsani, 2017; Karav et al., 2017; Kuhi et al., 2021). However, some *E. coli* have acquired specific virulence factors (VFs) that might help the bacteria to invade the mammary gland, survive, and multiply in milk (Blum et al., 2008, 2015, 2018; Olson et al., 2018; Salamon et al., 2020; Zhou et al., 2021), turning the mammary gland into an amenable opportunistic habitat for these bacteria. Given the relevance of mastitis caused by *E. coli*, this paper reviews the important aspects of the disease, the characteristic of the etiologic agent *E. coli*, diagnosis, and treatments.

## Clinical and Subclinical Mastitis

Based on disease symptoms, mastitis can be categorized into two different types, including clinical and subclinical. Clinical mastitis is characterized by visible abnormalities in the udder and milk (Oliveira et al., 2013). Physical examination of the affected mammary gland may reveal redness and pain upon palpation, swelling (edema), and induration (hardening) (Sepúlveda-Varas et al., 2016). Systemic symptoms and behavioral changes, including fever, anorexia, depression, lethargy, and a reduction in grooming might also occur (Medrano-Galarza et al., 2012; Dittrich et al., 2019). Cattle with clinical mastitis are also affected by poor milk quality, as evidenced by increased somatic cell count (SCC) and compositional changes in milk (Kayano et al., 2018; Malik et al., 2018; Skarbye et al., 2018). SCC is higher compared to the typical counts of under 200,000 cells/mL (Cobirka et al., 2020). The compositional changes in milk include the reduction of lactose content (Bezman et al., 2015; Costa et al., 2019), but little effect on fat and protein contents was observed (Kester et al., 2015; Tomazi et al., 2015). Clots and flakes can appear in milk, as well as clear serum-like or bloody discharges (Rainard and Riollet, 2006; Brandt et al., 2010). From 1996 to 2014, the reported incidence of clinical mastitis in United States dairy farms increased from 13% (USDA, 1996) to 25% (USDA, 2016). *E. coli* is one of the most common causes of clinical bovine mastitis, usually associated with acute symptoms such as dysstasia, diarrhea, and cool extremities (Bradley and Green, 2000; Blum et al., 2014; Hagiwara et al., 2014). *E. coli* causes predominantly acute infections, of short duration (10–30 days), mainly in the last 2 weeks of the dry period and in the first weeks postpartum, although reinfections may occur throughout lactation (Hogan and Smith, 2012; Wente et al., 2020). In most cases, *E. coli* is eliminated by the host immune response (Günther et al., 2017). However, severe cases with systemic involvement are difficult to treat and have a reserved prognosis (Günther et al., 2017).

Subclinical mastitis rarely poses an immediate threat to the animal’s life and is 15–40 times more common than clinical mastitis (Seegers et al., 2003). This form of the disease cannot be detected by visual inspection of the udder or milk since both appear normal (Ruegg, 2017). Consequently, due to the concealed nature of the disease, dairy farmers are unaware of the extent of milk quality loss and the risk of subclinical mastitis spreading to other cows in the herd. Subclinical mastitis is mostly caused by gram-negative bacteria, such as *E. coli*, *Klebsiella pneumoniae*, and *Serratia marcescens* (Schukken et al., 2012; Azevedo et al., 2016). These pathogens can be transmitted from an infected quarter to an uninfected quarter within the same animal or a different cattle by milking machine (e.g., teat cup liners), dirty milkers’ hands or gloves, and udder washcloths during the milking process (Klaas and Zadoks, 2018; Mohammed et al., 2020). Subclinical mastitis is the most important intramammary illness in cattle, and it costs the dairy farmer more than clinical mastitis due to the dramatic decrease in milk production and quality (Bobbo et al., 2017; Gonçalves et al., 2018).

## The Dilemma of Mammary Pathogenic *Escherichia coli* as a New Pathotype

Extraintestinal pathogenic *E. coli* (ExPEC) include different sub-groups, e.g., uropathogenic *E. coli* (UPEC), meningitis-associated *E. coli* (MNEC), and sepsis-associated *E. coli* (SEPEC), causing urinary tract infection, meningitis, peritonitis, and septicemia, respectively (Croxen and Finlay, 2010; Mellata, 2013; Biran and Ron, 2018). This *E. coli* pathotype-based classification is based on the site of infection, symptoms of the disease, and types of VFs. A novel ExPEC pathotype known as MPEC, one of the most common etiologic agents of bovine mastitis, has been suggested (Shpigel et al., 2008). Compared to other bacteria like *Campylobacter* spp*., Clostridium* spp., and *Ruminococcus* spp. (Zhang et al., 2017), MPEC have undergone evolutionary adaptations (e.g., acquisition of VFs) that improve its colonization of the unique environmental niches found within the mammary gland (Shpigel et al., 2008). These niches include competing microbes, soluble and cellular antimicrobials in milk, e.g., lactoferrin and lactoperoxidase (Kawai et al., 2015; Koshiishi et al., 2017), and the innate response elicited by immune cells (Wellnitz and Bruckmaier, 2012). Consequently, *E. coli* with specific VFs selected within the mammary gland are better suited to cause bovine mastitis (Blum et al., 2015; Richards et al., 2015; Goldstone et al., 2016; Roussel et al., 2017; Sun et al., 2021; Zhou et al., 2021).

Some research studies found that *E. coli* isolated from cows with mastitis is less genotypically diverse than the environmental strains, and they mostly lack known *E. coli* VFs (Ghanbarpour and Oswald, 2010; Blum and Leitner, 2013; Aslam et al., 2021). This lack of VFs not only reflects the genome plasticity of *E. coli* but, most importantly, it suggests that mastitis-associated *E. coli* may have undergone some selective pressure based on the presence of specific VFs associated with the ability to survive in milk (Blum et al., 2015; Goldstone et al., 2016). However, despite recent publications focusing on *E. coli* genomes isolated from mastitis-affected cattle (Blum et al., 2015, 2018; Richards et al., 2015; Goldstone et al., 2016; Kempf et al., 2016; Olson et al., 2018; Aslam et al., 2021; Sun et al., 2021), researchers were unable to agree upon a common genetic group of putative VFs to all MPEC isolates. Importantly, genome studies on mastitis-associated *E. coli* have been questioned by some investigators, who argue the need to simultaneously analyze the host-pathogen interaction (Zadoks et al., 2011; Leimbach et al., 2017). For example, it is important to associate physiological traits with genomic data, including techniques such as dual RNA-Seq of the host and bacteria (Westermann et al., 2016, 2017), Tn-Seq to test virulence association of genes *in vivo* (Opijnen et al., 2009; Peek et al., 2020), comparative SNP analysis of orthologous genes and intergenic regions (Rocha et al., 2019), proteomics (Zhang H. et al., 2018; Bathla et al., 2020), and metabolomics (Tong et al., 2019; Hu et al., 2021).

Several research studies suggest that *E. coli* isolated from mastitis-affected cattle are naturally occurring commensals from the gastrointestinal tract of bovine (Le Gall et al., 2007; Tenaillon et al., 2010; Tourret and Denamur, 2016), mostly from phylogroups A and B1 (Suojala et al., 2011; Blum et al., 2018; Olson et al., 2018; Zhang D. et al., 2018; Guerra et al., 2019, 2020; Aslam et al., 2021; Bag et al., 2021). In other words, it is more likely that *E. coli*’s VFs implicated in bovine mastitis have their principal function in the colonization and survival in the highly diverse gastrointestinal environment (Leimbach et al., 2017). Consequently, *E. coli* isolated from mastitis-affected cattle are more likely to be opportunistic and might infect only cattle with a deficient immune system, particularly during the peripartum when leukocyte recruitment to the mammary gland is reduced (Leblanc, 2020). In this case, environmental variables and the cattle predispositions (e.g., innate immune system related to the health status, stage of lactation, and parity) (Burvenich et al., 2003; Wenz et al., 2006) are more important than the invading *E. coli*’s VFs in determining the severity of mastitis.

Table 1 shows studies on different virulence genes and VFs examined in *E. coli* isolates from bovine mastitis. Various virulence-related features have been suggested to explain the pathogenicity of *E. coli* in bovine mastitis, including but not limited to the activation of the innate immune system by pathogen-associated molecular patterns (PAMPs) (Schukken et al., 2011; Bhattarai et al., 2018; Petzl et al., 2018), resistance to serum (Blum and Leitner, 2013; Guerra et al., 2020; Aslam et al., 2021) and neutrophil killing (Kaipainen et al., 2002; Roussel et al., 2017), adhesion and invasion of mammary epithelial cells (Dogan et al., 2006, 2012), and survival and proliferation in milk though secretion system (Richards et al., 2015; Sun et al., 2021). However, it is important to note that although the aforementioned virulence-associated properties benefit *E. coli* to thrive in the mammary gland environment, they are not unique to mastitis-associated *E. coli*, but are also common in ExPEC. Moreover, *E. coli* strains isolated from cattle with mastitis usually do not present the same virulence characteristics, demonstrating the high genetic abundance and variability of mastitis-causing *E. coli*.

**TABLE 1:** Studies on different virulence genes examined in *E. coli* isolates from bovine mastitis.

Detected virulence genes (associated VFs/Function)	References
*iucD*, *traT*	Aslam et al., 2021
type VI secretion system, type IV secretion system, type IV pili, *hlyA*, *cnf2*	Sun et al., 2021
*fimH, ecpA, fimA, traT, ompT, irp2, hlyA*	Guerra et al., 2020
*fecIRABCDE*	Blum et al., 2018
*ompC*, *ompF*, *fimH*, *colV*, *irp2*, *fyuA*, *eaeA*, *ler*, *iucD*	Zhang D. et al., 2018
*epr1* (type III secretion system)	Leimbach et al., 2017
*fecA*, *lpfA*, *Ipx*, *ecp*	Kempf et al., 2016
*f17A*, *irp2*, *astA, iucD*, *colV*	Liu et al., 2014
*lpfA*, *iss*, *astA*	Blum and Leitner, 2013
*stx1*, *stx2*, *eaeA*, *f41*	Momtaz et al., 2012
*irp2*, *iucD, papC*, *iss*, *cva*, *afa8*, *astA*, *f17*, *cnf2*, *eaeA*, *vat*, *sfaD*, *tsh*, *saa*	Suojala et al., 2011
*f17*, *eaeA*	Momtaz, 2010
*eaeA, cnf1*, *cnf2*, *cs31A*	Wenz et al., 2006
*stx1*, *cnf2*, *vt2e*, *eaeA*	Bean et al., 2004
*traT, cnf1, cnf2, aer*, *f17, sfa*, *pap, afa8D, afa8E*	Kaipainen et al., 2002

*iucD, irp2 = genes encoding siderophore, traT = gene encoding serum resistance, fimH and fimA = gene encoding type 1 pili, ecpA = gene encoding E. coli common pilus, tratT and ompT = gene encoding serum resistance, hlyA = gene encoding hemolysin, fecIRABCDE = genes encoding ferric dicitrate uptake, ompC and ompF = genes encoding outer membrabe porin proteins, ler = gene encoding intimin, colV = gene encoding plasmid, eaeA = gene encoding intimin, iucD = gene encoding siderophore, erp1 = gene encoding type lll secretion system, fecA = gene encoding iron acquisition system, lpfA = gene encoding long polar fimbriae, Ipx = gene encoding lipid A synthesis, ecp = gene encoding type VI secretion system, f17 = gene encoding fimbria, irp2 = gene encoding yersinia bactin, astA = gene encoding the heat-stable toxin, iss = gene encoding serum resistance, stx1 and stx2 = gene encoding verotoxin, f41 = gene encoding fimbriae, pap and papC = genes encoding fimbria, cva = gene encoding plasmid, afa8, afa8D, and afa8E = genes encoding afimbrial adhesin, cnf1 and cnf2 = gene encoding necrotizing cytotoxic factor, vat = gene encoding toxin, sfa and sfaD = genes encoding fimbria, tsh = gene encoding hemagglutinin, saa = gene encoding adhesin, cs31A = gene encoding adhesin, vt2e = gene encoding verotoxin, aer = gene encoding aerobactin and fyuA = gene encoding the yersiniabactin receptor.*

Interestingly, the iron scavenging by the ferric dicitrate system has been shown to be strongly associated with mastitis-associated *E. coli* (Blum et al., 2015; Goldstone et al., 2016; Kempf et al., 2016; Aslam et al., 2021). The *Fec* system is essential for the growth of *E. coli* in milk and may influence disease severity in infections (Olson et al., 2018). Iron in bovine milk is limited and kept at quantities below those required to enable *E. coli* growth because most iron is attached to citrate (Blum et al., 2018) and a lesser amount to lactoferrin (Karav et al., 2017), transferrin (Kupczyński et al., 2017), xanthine oxidase (Harrison, 2006), and certain caseins (Usami et al., 2011). To overcome the low amount of free iron in milk, *E. coli* expresses a variety of iron transport systems, such as the high-affinity siderophores (e.g., enterobactin, aerobactin, ferrichrome) and iron-regulated outer membrane proteins (IROMP) (van der Helm, 1998) that bind to ferric siderophore complexes for transportation into the bacterial cell. In a population-level genomic analysis, Blum et al. (2018) showed that the *Fec* system is required for the pathogenicity of *E. coli* in the udder environment, as the prototypical MPEC strain P4 was unable to induce mastitis without the system, and the non-mammary gland-pathogenic K71 strain gained MPEC capability with its addition. Importantly, while additional research is needed to fully comprehend the significance of the *Fec* system in mastitis-associated *E. coli*, this critical structural component is a promising target for novel chemotherapeutics, enhanced mastitis therapy medicines, and novel vaccines.

### Virulence Attributes of *Escherichia coli* Associated With the Severity of Mastitis

The impact of known virulence genes on the severity of bovine mastitis caused by *E. coli* is currently unclear (Zadoks et al., 2011), and numerous virulence gene profiles have been found in both persistent and transient isolates, suggesting a high genotypic variability of mastitis-associate *E. coli* (Ghanbarpour and Oswald, 2010; Fernandes et al., 2011; Dogan et al., 2012; Fairbrother et al., 2015). Importantly, clinical symptoms of *E. coli* mastitis may be related to genes that have yet to be discovered or to characteristics related to the host (e.g., stage of lactation, number and function of circulating neutrophils, SCC, age of the cattle, nutritional and metabolic status, and genetic resistance) (Burvenich et al., 2003). However, persistent infections seem to be related to particular traits (Lippolis et al., 2014), especially the ability of *E. coli* to transfer from milk to mammary epithelial cells and survive intracellularly (White et al., 2010). Epithelial cell adhesion and invasion are important events in chronic or persistent infection (Finlay and Cossart, 1997). *In vitro* studies demonstrated that persistent mastitis-associated *E. coli* strains have a higher ability to adhere, invade and survive in bovine mammary epithelial cells line MAC-T than transient strains (Dogan et al., 2006), are more capable of penetrating an endosome-like compartment of mammary epithelial cells (Passey et al., 2008), and have a different intracellular trafficking mechanism than transient strains (Almeida et al., 2011). Moreover, it was shown that persistent *E. coli* isolates were more likely to carry lpf genes (encodes for long polar fimbriae), which correlates with *E. coli*’s ability to invade mammary epithelial cells (Dogan et al., 2012). Interestingly, in a host-pathogen gene expression study conducted by Kerro Dego et al. (2012), both acute and persistent *E. coli* mastitis strains boosted immune response gene expression (e.g., IRF1, CD83, IL-1α, IL-6, IL-8, CCL20, CXCL 2, MHC 1, Casp 3, CFB), but the immune response increase was substantially greater in cells cocultured with the acute *E. coli* strain than in cells cocultured with the persistent strain. Similarly, other investigations reported greater up-regulation in immune response functions, inflammation, and antigen processing and presentation in the acute phase than in the chronic phase of bovine mastitis (Mitterhuemer et al., 2010; Rinaldi et al., 2010; Buitenhuis et al., 2011). The induction of a modest immune response by persistent strain is insufficient to eradicate the infection, allowing *E. coli* to survive in the mammary gland for a lengthy period.

## Host Immune Response to *Escherichia coli*

### Innate and Acquired Immunity of the Mammary Gland

Bovine innate immunity comprises physical barriers at the tip of the teat and elements such as neutrophils, macrophages, cytokines, natural killer (NK) cells, lactoferrin, and complement, that act predominantly in the early stages of *E. coli* infections. This variety of immune cell types constitutes the somatic cells (SC), with neutrophils being the predominant type at the beginning of inflammation, representing about 90% of the SC increase (Ingvartsen and Moyes, 2015; Becheva et al., 2017). When pathogens invade the mammary gland, they face the first line of cellular defense, such as phagocytosis by neutrophils, which usually respond promptly to the inflammatory process (Mehrzad et al., 2004; Aitken et al., 2011; Bassel and Caswell, 2018). As a result, the severity of mastitis is determined by the speed with which neutrophils are recruited to the site of infection and how effectively this cell type phagocytes the pathogen (Mehrzad et al., 2004; Alhussien et al., 2016; Chengolova et al., 2021). Importantly, the efficacy of the innate response influences the incidence of new intramammary infections (Sordillo, 2018), clinical severity (Pezeshki et al., 2011), and duration of the case (Blum et al., 2017). Another crucial component of the host’s innate immunity is lactoferrin, an iron-tropic glycoprotein with bacteriostatic properties produced by epithelial cells and leukocytes (Shimazaki and Kawai, 2017). In ruminants, lactoferrin has been shown to inhibit the multiplication of *E. coli* by binding to iron ions, making them unavailable for bacterial growth (Rybarczyk et al., 2017; Kieckens et al., 2018). Additionally, cytokines including interleukins (IL), colony-stimulating factor (CSF), interferon (IFN), and tumor necrosis factor (TNF) are crucial in the mammary gland innate defense (Bhattarai et al., 2018; Vitenberga-verza et al., 2022). Cytokines are responsible for local signs of inflammation such as swelling, redness, pain, and systemic symptoms such as fever, tachycardia, increased respiratory rate, anorexia, and depression (Sordillo et al., 1997; Thompson-Crispi et al., 2014). When innate immunity is not efficient in fighting the infection, the specific (or acquired) immunity mediated by lymphocytes occurs. In response to the inflammation, antibodies produced by lymphocytes pass from the bloodstream to the milk due to increased vascular permeability of the gland. The inflammatory response to mastitis aims to eradicate the microorganism responsible for the infection, neutralize the pathogen’s toxins, and then regenerate injured udder tissue so that the volume of milk normally produced is quickly recovered.

### Mammary Pathogenic *Escherichia coli*-Elicited Immune Response

**FIGURE 1:**
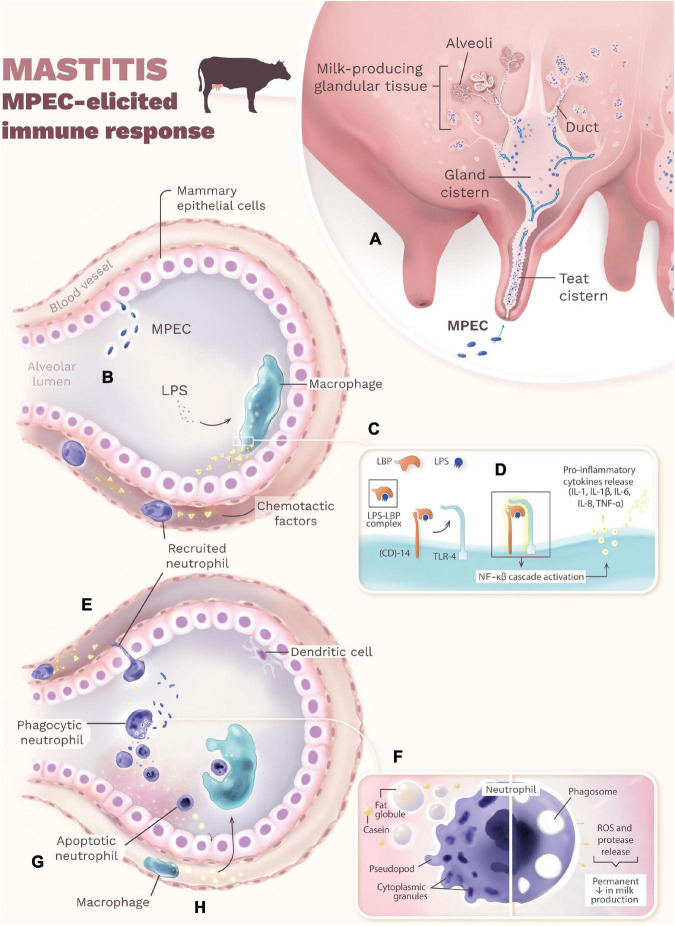
The onset of mastitis occurs when MPEC penetrates through the teat canal, multiplies in the teat and gland cisterns, and spreads throughout the milk-producing glandular tissue **(A)**. The multiplication and lysis of MPEC release LPS from the bacteria’s outer membrane **(B)**. LPS binds to the LBP, and the LPS-LBP complex binds to the CD-14, which leads the LPS to a TLR-4 on the surface of macrophage **(C)**. When LPS binds to its respective TLR, an intracellular signaling cascade is activated (NF-κβ), which induces macrophages and mammary epithelial cells to synthesize pro-inflammatory cytokines and acute-phase proteins **(D)**. Chemotactic factors, especially IL-8, then recruit neutrophils from the bloodstream that function as phagocytes at the site of infection in the alveoli **(E)**. The exposure of neutrophils to fat globules and casein leads to loss of cytoplasmic granules (reduced bactericidal activities) and altered morphology (rounding), eliminating pseudopods needed for phagocytosis **(F)**. During phagocytosis, neutrophils release chemicals that eliminate MPEC but also injure mammary epithelial cells, resulting in a permanent decrease in milk production **(F)**. To minimize mammary tissue damage, neutrophils undergo programmed cell death (apoptosis) and release chemokines that attract macrophages to the site of infection (Paape et al., 2003) **(G)**. In the last step of the inflammatory reaction, macrophages quickly phagocytose apoptotic neutrophils (efferocytosis), minimizing the release of granular contents that damage the tissue **(H)**. Interestingly, whereas in the mammary alveolar, dendritic cells are generally non-responsive to MPEC infection.

The onset of mastitis occurs when MPEC penetrates through the teat canal, multiplies in the teat and gland cisterns, and spreads throughout the milk-producing glandular tissue (Figure 1A). Once inside the mammary gland, MPEC ferment lactose (Blum et al., 2008; Lippolis et al., 2009) and use it as an energy source to multiply without adhering to the gland’s epithelial surface, remaining in the cisterns, ducts, and alveolar lumen (Roussel et al., 2017; Bianchi et al., 2019). Some MPEC strains can adhere to the udder epithelium, causing chronic intramammary infections and recurrent cases of clinical mastitis (Dogan et al., 2006; Roussel et al., 2017). The multiplication and lysis of MPEC release LPS from the bacteria’s outer membrane (Figure 1B). LPS binds to the LPS binding protein (LBP), and the LPS-LBP complex binds to the cluster of differentiation (CD)-14, which leads the LPS to a toll-like receptor-4 (TLR) on the surface of macrophages (Bhattarai et al., 2018; Eckel and Ametaj, 2020; Figure 1C). When LPS binds to its respective TLR, an intracellular signaling cascade is activated, including the activation of nuclear factor kappa-beta (NF-κβ), which induces macrophages and mammary epithelial cells to synthesize pro-inflammatory cytokines [e.g., interleukin (IL)-1β, IL-1, IL-6, IL-8, and tumor necrosis factor (TNF-α)] and acute-phase proteins (e.g., serum amyloid A and haptoglobin) (Fitzgerald et al., 2007; Porcherie et al., 2012; Roussel et al., 2015; Günther et al., 2017; Curone et al., 2018; Akhtar et al., 2020; Bronzo et al., 2020; Khan et al., 2020; Figure 1D). Importantly, TNF-α is the main mediator of endotoxic shock that occurs in cases of super-acute mastitis caused by coliforms (Hogan and Smith, 2003; Shaheen et al., 2020).

Chemotactic factors, especially IL-8, recruit neutrophils from the bloodstream that function as phagocytes at the site of infection in the alveoli (Zaatout, 2022; Figure 1E). The efficiency of neutrophils’ diapedesis and phagocytosis directly influence the evolution of the local inflammatory process and the severity of mastitis (Elazar et al., 2010; Roussel et al., 2017; Bassel and Caswell, 2018). Although opsonization is not essential for phagocytosis, immunoglobulins (Ig) such as IgG2 and IgM recognize MPEC through Fab-regions and bind to neutrophils *via* Fc-receptors on the plasma membrane, leading to phagocytosis in a “zipper mechanism” (Burvenich et al., 2003). The exposure of neutrophils to fat globules and casein leads to loss of cytoplasmic granules (reduced bactericidal activities) and altered morphology (rounding), eliminating pseudopods needed for phagocytosis (Paape et al., 2003; Swain et al., 2014; Sordillo, 2018; Panda et al., 2019; Figure 1F). Consequently, many neutrophils are necessary to cope with the reduced phagocytic capabilities and control the infection. During phagocytosis, neutrophils release chemicals, e.g., reactive oxygen species (ROS) (Lauzon et al., 2005) and proteases (Mehrzad et al., 2005), that eliminate MPEC but also injure mammary epithelial cells, resulting in a permanent decrease in milk production (Zhao and Lacasse, 2008; Eckel and Ametaj, 2020; Figure 1F). To minimize mammary tissue damage, neutrophils undergo programmed cell death (apoptosis) and release chemokines that attract macrophages to the site of infection (Sladek et al., 2005; Figure 1G). In the last step of the inflammatory reaction, macrophages quickly phagocytose apoptotic neutrophils (efferocytosis), minimizing the release of granular contents that damage the tissue (Greenlee-Wacker, 2016; Figure 1H); whereas in the mammary alveolar, dendritic cells are generally non-responsive to MPEC infection. However, when efferocytosis done by macrophages is ineffective, there is increased clearing of apoptotic neutrophils by dendritic cells (Tian et al., 2016). Curing of the mammary gland is only successful if minimal tissue damage occurs (Gonen et al., 2007).

## Bovine Mastitis Diagnosis

Clinical mastitis is easily detected by visible abnormalities in the udder (e.g., redness, swelling, increased temperature, and pain upon physical palpation) and milk (e.g., presence of clots to clear, serum-like, or bloody secretions). In subclinical mastitis, however, indirect inflammation measures must be used to detect the disease. Contrarily to clinical mastitis, the only visible sign of animals with subclinical mastitis is reduced milk production (Bobbo et al., 2017; Martins et al., 2020). Consequently, the diagnostic requires complementary tests based on the SCC of milk (Adkins and Middleton, 2018). The SCC is used as an indicator of mammary gland health and milk quality. Usually, healthy cows produce milk with less than 100,000 SCC/mL, while cows with mastitis have a minimum SCC of 200,000 SCC/mL (Kelly et al., 2011). The California Mastitis Test (CMT) estimates the SCC on milk and can be performed at the cow-side to detect mastitis (Bhutto et al., 2012; Godden et al., 2017). Electronic SCC is another test performed on the total mixed (composite) milk from all quarters of each cow, and it helps track the progress of control programs (Alhussien and Dang, 2018). Microbiologic culture of milk from individual quarters or composite samples of individual animals is another tool to identify *E. coli* involved in clinical mastitis (Royster et al., 2014; Ferreira et al., 2018). However, since the number of bacteria may be too small to be identified by standard methods, bacteria are not detected in roughly 30% of samples (Taponen et al., 2009; El-Sayed et al., 2017; Singh et al., 2018). Repeated culture and specialized culture techniques may be required in these cases.

## Improved Diagnosis of *Escherichia coli* Mastitis: Challenges and Future Approaches

Because of the high costs of *E. coli* mastitis and the importance of maintaining cattle’s health and well-being, early diagnosis is critical. As previously mentioned in Section “Bovine Mastitis Diagnosis,” SCC and CMT are techniques commonly used to diagnose bovine mastitis. Unfortunately, both techniques have drawbacks, including subjective interpretation of CMT results (Swinkels et al., 2021), which could result in false positives and negatives (Fosgate et al., 2013), and factors such as lactation number, milk yield, stress, season, and breed that could influence with SCC’s interpretation (Alhussien and Dang, 2018). As a result, genotypic approaches are also used to supplement phenotypic identification of bovine mastitis, providing a viable option for resolving issues such as false-negative results. For example, approximately 30% of clinical mastitis samples do not grow in bacterial culture (Taponen et al., 2009); however, polymerase chain reaction (PCR) is sensitive to detect growth-inhibited and reduces false-negative results (El-Sayed et al., 2017). Other molecular typing methods used for the identification of bovine mastitis pathogen at the species level include ribotyping (Zadoks et al., 2011) and amplified fragment length polymorphism (AFLP) (Leung et al., 2004); at the strain level, include restriction fragment length polymorphism (RFLP) (Ombarak et al., 2018), pulsed-field gel electrophoresis (PFGE) typing (Freitag et al., 2017), and multiple-locus variable-number tandem repeat analysis (MLVA) (Elsayed et al., 2021); and at both species and strain levels include transfer DNA intergenic spacer length polymorphism (Chakraborty et al., 2019) and DNA sequencing of housekeeping genes (Ali et al., 2017). Another genotypic method is microarray technology, which can detect seven common species of mastitis-causing pathogens in as little as 6 h, with a sensitivity of 94.1% and a specificity of 100% (Lee et al., 2008).

New biomarkers for high sensitivity and specificity, rapid and efficient with a “cow-side” application, are among the development prospects for novel *E. coli* mastitis diagnosis techniques (Duarte et al., 2015; Sharifi et al., 2018; Li L. et al., 2019). For example, proteomic research has yielded data on protein expression patterns that can be used to find novel therapeutic targets (e.g., bacterial immunogenic proteins for vaccines) and diagnostic biomarkers for bovine mastitis (Ceciliani et al., 2014). Importantly, a detection method’s suitability for routine mastitis diagnosis is determined by its specificity, sensitivity, cost, processing time, and ability to handle a large number of milk samples. Proteomic technique for accurate biomarkers is feasible for early identification of mastitis and treatment efficacy, as well as the discovery of possibly novel targets for alternative therapy development. These advancements, however, are still not suitable for routine diagnosis of bovine mastitis.

## Bovine Mastitis Treatment and Control

Since mastitis results from the interaction of many different factors, especially milking procedures and cleanliness of the environment, there is no single way to treat and control the disease. The control of environmental mastitis is especially challenging because *E. coli* is ubiquitous in the cattle’s environment. Thus, good herd health management and environmental hygiene are the most critical factors in preventing this type of mastitis (Klaas and Zadoks, 2018; Zigo et al., 2021). Except for severe infections during the puerperal period, antibiotic therapy for coliform mastitis is not recommended due to the high spontaneous cure rates (Suojala et al., 2013; Ruegg, 2017) and the risk of antibiotic contamination of the bulk milk (Tang et al., 2017). Supportive care such as fluid therapy and treatment with steroidal or non-steroidal anti-inflammatory drugs (e.g., dexamethasone) should be the first treatment option (Barlow, 2011; Kayitsinga et al., 2017; Sintes et al., 2020). Parenteral administration of fluoroquinolones (e.g., enrofloxacin, danofloxacin, marbofloxacin) or third-generation cephalosporin (e.g., ceftiofur) is recommended to treat severe *E. coli* mastitis (Suojala et al., 2013; Krömker and Leimbach, 2017).

Another approach to prevent coliform mastitis is to boost the animal’s specific immunity through vaccination (Ismail, 2017; Rainard et al., 2021a), particularly with the *E. coli* J5 vaccine (Suojala et al., 2013; Pomeroy et al., 2016; Herry et al., 2017). While the teat canal is the first line of defense against intramammary infections, milk neutrophils are the major immune defense when *E. coli* has already invaded the teat canal (Sordillo, 2005; Lind et al., 2015; Bassel and Caswell, 2018). When intramammary infections are not quickly controlled by neutrophils, allowing LPS and lipoproteins to escape into the bloodstream, antibodies in the blood serum must be present to neutralize these potent toxins. Thus, preventing coliform mastitis through vaccination aims to achieve high levels of effective anti-coliform antibodies and antitoxins, both in milk and in the bloodstream, and around the teat canal (Erskine et al., 2007; Wilson et al., 2009; Kawai et al., 2020). Two mutant strains of bacteria, *E. coli* O111:B4 (J5) and *Salmonella* Typhimurium Re-17 have been used in commercial preparations to immunize cows against coliform mastitis (Erskine, 2012; El-Sayed and Kamel, 2021). *E. coli* O111:B4 (J5) is a rough mutant strain with an incomplete “O” polysaccharide chain in the cell wall, exposing the homologous half of the LPS core antigen (Zaatout, 2021). When this antigen is exposed, it stimulates the synthesis of immunoglobulins (e.g., IgM, IgG_1_, IgG_2_) that cross-react with other bacteria’s core antigens, resulting in immunity and protection against a wide range of bacterial genera and strains (Rainard et al., 2021a,b). The J5 vaccination boosts blood and milk titers of specific antibodies against *E. coli* LPS, enhancing its opsonization (Brade et al., 2013; Steele et al., 2019). Importantly, neutrophils can phagocyte bacteria more effectively when pathogens are opsonized by antibodies (Rainard et al., 2022). Vaccinations are often timed to correspond with the highest risk of acquiring mastitis by coliforms (e.g., pre- and immediate postpartum), with immunizations being carried out 30 days before delivery and in the first week following delivery (Rainard et al., 2021a). Increasing the cattle’s natural ability to fight infections in the mammary gland during periods of increased susceptibility is one alternative for reducing mastitis in dairy cows.

The outer membrane protein A (OmpA) has also been considered a potential antigen in the bovine mastitis vaccine associated with *E. coli* (Rainard et al., 2017; Chen et al., 2020; Liu et al., 2021). OmpA is a crucial virulence factor in the pathogenesis of *E. coli*, playing a key role in pore and biofilm formation, host cell invasion, and multidrug resistance (Confer and Ayalew, 2013). For example, recombinant OmpA protein fragments could regulate the expression of cytokines, chemokines, nitric oxide synthase, and cyclooxygenase-2, preventing *E. coli* meningitis in mice (Hsieh et al., 2016). Moreover, OmpA has been found to reduce complement-dependent or phagocytic-dependent killing of OmpA-positive *E. coli* (Krishnan and Prasadarao, 2012). Therefore, OmpA is a potential antigen in vaccine development and for the prevention of *E. coli* infection. However, despite current investigations on mastitis vaccine, there is limited information on OmpA as a viable vaccine antigen in mastitis-associated *E. coli.* Among the few studies on OmpA as a vaccine candidate in *E. coli* mastitis, Rainard et al. (2017) investigated the immunogenicity of OmpA by immunizing cows with a recombinant protein (rEcOmpA) to potentially stimulate antibodies and cell-mediated immune responses. Interestingly, the intramammary immunization elicited antibodies (e.g., IgG_1_ and IgG_2_), but the antibodies could not interact with the mastitis-causing strain P4, implying that they were unable to reach the accessible regions of OmpA molecules located at the outside leaflet of the bacteria external membrane. Collectively, the aforementioned studies show that the potential of OmpA as a vaccine to prevent *E. coli*-associated bovine mastitis has yet to be determined.

## Antimicrobial Resistance of Mastitis-Associated *Escherichia coli*

Antibiotics are crucial in curing serious cases of bovine mastitis caused by *E. coli* (Bengtsson et al., 2009; Suojala et al., 2013). Several antimicrobial agents have been approved in the United States to treat *E. coli* mastitis, such as tetracyclines, macrolides, sulphonamides, quinolones, and β-lactams (Mathew et al., 2007; Bengtsson et al., 2009). Unfortunately, antibiotic therapy has frequently been demonstrated to have little or no effect in treating clinical or subclinical mastitis caused by coliforms in cattle (Suojala et al., 2010; Ruegg, 2018). This fact is evidenced by the increase in antimicrobial-resistant isolates (Skočková et al., 2015; Zhang D. et al., 2018; Cao et al., 2019; Ngaywa et al., 2019; Yu et al., 2020; Majumder et al., 2021) and the growing number of recurrent and persistent mastitis cases (Fairbrother et al., 2015; Bag et al., 2021; Taniguchi et al., 2021), often caused by the same *E. coli* isolate. *E. coli* isolated from the milk of mastitis-affected cattle is resistant to numerous antibiotic classes, including but not limited to aminopenicillin (e.g., cloxacillin) (Holko et al., 2019; Guerra et al., 2020), polypeptide (e.g., bacitracin) (Tanih et al., 2015), lincosamide (e.g., lincomycin) (Fazel et al., 2019), and macrolide (e.g., erythromycin) (Yu et al., 2020; Ahmed et al., 2021). In clinical mastitis, additional caution is advised when determining whether or not to treat cattle with antimicrobials, as antibiotics are not always essential, such as in cases of negative cultures and minor coliform infections (Makovec and Ruegg, 2003; Suojala et al., 2011; Oliveira and Ruegg, 2014; Fuenzalida and Ruegg, 2019). However, severe cases of mastitis caused by *E. coli* must be treated immediately following diagnosis due to endotoxin-induced shock (Persson et al., 2015).

Pathologic alterations in the mammary gland, such as necrosis and ischemia, might obstruct antimicrobial distribution in the udder parenchyma, preventing the antimicrobial from reaching the site of infection or reaching sub-inhibitory concentrations (Akers and Nickerson, 2011). Importantly, the ability of *E. coli* to form biofilms on the tissue surface of the infected mammary gland, thereby establishing an inherent resistance to several antimicrobials treatment, is one of the hypotheses to explain the emergence of recurring mastitis (Olson et al., 2002; Melchior et al., 2006; Pedersen et al., 2021; Rudenko et al., 2021). *E. coli* is notorious for being a reservoir for antimicrobial resistance genes, and it has the ability to horizontally transmit such genes to other pathogenic bacteria (Freitag et al., 2017; Zhang et al., 2020). Investigations have shown that the most prevalent resistance genes found in mastitis-associated *E. coli* encode resistance to aminoglycoside (e.g., *aadA*), streptomycin (e.g., *strA*, *strB)*, tetracycline (e.g., *tetA*, *tetC*), sulfonamide (e.g., *sulI*, *sulII*), ampicillin (e.g., *ampC*), and β-lactams (e.g., *blaTEM*, *blaCTX-M*) (Srinivasan et al., 2007; Freitag et al., 2017; Todorović et al., 2018; Fazel et al., 2019; Yu et al., 2020). Importantly, the extended-spectrum β-lactamase enzymes (ESBL) are a major concern in the antibiotic resistance mechanism of *E. coli* mastitis (Schmid et al., 2013; Klaas and Zadoks, 2018; Yang et al., 2018). These enzymes are located in plasmids and can hydrolyze penicillin (Padmini et al., 2017), third and fourth-generation cephalosporins (Li Q. et al., 2019), and monobactam antibiotics (Jacoby, 2009; Bush and Jacoby, 2010). The detection of ESBL-producing isolates is of concern due to the direct association between multi-resistant bacteria and high mortality rates in humans (Bengtsson et al., 2009). There is a clear increase in the prevalence of ESBL *E. coli* intestinal carriage among healthy individuals worldwide (2.6% in 2003–2005 to 21.1% in 2015–2018; an average increase of 1.2% per year) (Bezabih et al., 2021). Importantly, several countries, including China (Ali et al., 2016), Egypt (Ahmed et al., 2021), Germany (Eisenberger et al., 2018), and Greece (Filioussis et al., 2020), have been reporting the occurrence of ESBL-producing *E. coli* in cattle with mastitis. Moreover, the presence of ESBL-producing *E. coli* in milk raises concerns about the risks of ingesting unpasteurized milk and dairy products, potentially transmitting ESBL-producing *E. coli* to humans (Freitag et al., 2017). The prevalence of these isolates in milk has ranged from 0.4% (Freitag et al., 2017) to 25.4% (Dahmen et al., 2013).

Tests used to verify the antimicrobial sensitivity of pathogens causing mastitis, including antibiogram, disc diffusion technique, and minimum inhibitory concentration (MIC), are alternatives for the rational use of antimicrobials, aiming to support the treatment only of quarters infected by agents with the possibility of cure. Nowadays, laboratories’ most often used method is the MIC, which produces more accurate findings than the antibiogram (Cheng et al., 2019; Bolte et al., 2020). This technique, however, does not yield reliable results when assessing sessile cells since sensitivity studies on bacteria organized in biofilms reveal that the concentration of antibiotics necessary for biofilm eradication is several times larger than that determined by MIC (Li et al., 2020; Pedersen et al., 2021).

## Conclusion

Under field conditions, colonization of the mammary gland is a polymicrobial event. Since milk is an excellent growth medium, many species of bacteria grow on it, colonize the mammary gland, and elicit inflammation and disease. Nevertheless, most mastitis cases are attributed to a single bacterial species or even to a single strain or clone of a species. The ability of *E. coli* to acquire exogenous DNA, including virulence genes, contributes to the development of pathogenic strains adapted to the mammary gland and potentially cause mastitis. MPEC has been proposed as a new pathotype responsible for causing mastitis in dairy animals. Some mastitis-associated *E. coli* strains evolved mechanisms that enable them to compete with other bacteria co-infecting the gland and evading the protective mechanisms of the host. However, it is important to note that although the aforementioned virulence-associated properties benefit *E. coli* to thrive in the mammary gland environment, they are not unique to mastitis-associated *E. coli*, but are also common in ExPEC. It is crucial to understand the principles behind the infection strategy of *E. coli* so that new approaches to treat and prevent mastitis by this bacteria and perhaps other mammary pathogens can be developed. The use of antibiotics in farm animals and specifically to treat mastitis is gradually precluded, and thus new treatment and prevention strategies are needed.

## Author Contributions

DG and MM wrote the manuscript. DG developed figure and table. MM revised manuscript. Both authors contributed to the article and approved the submitted version.

## Conflict of Interest

The authors declare that the research was conducted in the absence of any commercial or financial relationships that could be construed as a potential conflict of interest.

## Publisher’s Note

All claims expressed in this article are solely those of the authors and do not necessarily represent those of their affiliated organizations, or those of the publisher, the editors and the reviewers. Any product that may be evaluated in this article, or claim that may be made by its manufacturer, is not guaranteed or endorsed by the publisher.
